# Subcutaneous Emphysema Induced by Cryotherapy: A Complication due to Previous Punctures

**DOI:** 10.1155/2015/374817

**Published:** 2015-06-10

**Authors:** Jared Martínez-Coronado, Bertha Torres-Álvarez, Juan Pablo Castanedo-Cázares

**Affiliations:** Department of Dermatology, Hospital Central “Dr. Ignacio Morones Prieto”, Universidad Autonoma de San Luis Potosí, 2395 Venustiano Carranza Avenue, 78210 San Luis Potosí, SLP, Mexico

## Abstract

Cryosurgery is a common therapeutic modality used in dermatology; therefore we must be aware of its possible adverse effects. We report a case of a patient with subcutaneous emphysema which occurred following the application of cryotherapy after multiple punctures of local anesthetic and intralesional steroids in a chest keloid scar. Despite the fact that this condition was gradually resolved after expectant observation, we warn about this complication when sprayed cryotherapy is preceded by multiple punctures on cutaneous lesions above bony surfaces. In similar settings, cryotherapy must be first administered or a cotton-tip applicator should be used.

## 1. Introduction

Modern cutaneous cryosurgery was introduced in the 1960s [1], since then it is commonly used by most dermatologists around the world. It is recognized that this treatment was first applied in 1974 for keloidal scars by Pirece [2]. Cryotherapy induces vascular damage that leads to anoxia and tissue necrosis reducing the keloidal scar thickness [2, 3]. Thus, it is not an innocuous treatment and dermatologist must be aware of its side effects which can be immediate or delayed. Frequent short-term adverse features include pain, syncope, hemorrhage, edema, blistering, fever, infection, and pyogenic granuloma [1, 3]; long-term changes consist in permanent hypo- or hyperpigmentation, pseudoepitheliomatous hyperplasia, milia, nerve damage, alopecia, scar formation, and cartilage necrosis [1, 3]. We report a patient with keloid scar who presented subcutaneous emphysema after cryotherapy application, an uncommon complication finding in dermatologic literature [4–7].

## 2. Case Report

**Figure 1:**
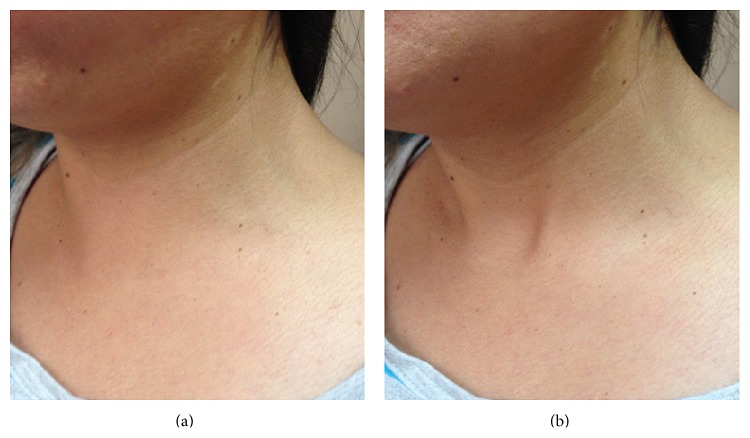
At (a) local subcutaneous augmented volume in the upper frontal chest and lower neck. (b) Normalization of the swelled area after one hour of conservative treatment.

A 28-year-old woman presented with an 18-month history of 2 × 10 cm keloid scaring induced by acne vulgaris on the upper frontal thorax. Her lesion was first locally anesthetized with intralesional lidocaine and afterwards infiltrated with acetonide of triamcinolone, followed by two 40-second cycles of sprayed cryotherapy. The patient came back to our facilities 30 minutes after the procedure because the upper area of the treated zone started to bulk. Physical examination only revealed swelling and cutaneous crepitus on palpation. There was no erythema or pain, nor local increased temperature. Vital signs were normal and there were no systemic symptoms other than minor anxiety triggered by this outcome. We made clinical diagnosis of subcutaneous emphysema as a complication of cryotherapy due to the timeline of the clinical history: punctures followed by sprayed cryotherapy and then a prompt presence of local subcutaneous emphysema. The patient was retained in our facilities and after one hour of observation the skin became normal in appearance. However, subcutaneous crepitation continued upon complete resolution after three days. Clinical changes are shown in Figure 1.

## 3. Discussion

Subcutaneous emphysema (SE) is defined by the presence of air or other gases within the soft tissue compartment [4, 5, 8]; it can be further divided as a result of infectious or noninfectious causes [5, 6]. There are several dermatologic conditions clustered within the second group such as irrigation of wounds with hydrogen peroxide, punch biopsy, or cryosurgery [6, 8]. During sprayed cryotherapy an opening on the skin surface acts as a one-way valve through which the positive pressure gas enters and spreads along the subcutaneous compartment [1, 4, 6]. Risk factors for SE secondary to cryotherapy are usually related to elderly patients. In these cases, atrophic skin is easier to be disrupted while applying positive pressure of a handheld spray device [5, 9]. This technique of cryotherapy on ulcerated skin after curettage procedures or freshly closed wounds has also been associated to this complication [4, 9]. Although this unfavorable event depends on the site where cryotherapy is applied, it is most likely to occur in areas of lax and thin skin, such as the periorbital area or on the dorsum of hands [1].

In our young patient, the SE was caused by the entrance of the sprayed liquid nitrogen through the disrupted skin, following the approximately 8 consecutive 27-gauge needle punctures performed on the treated area. The association between cryotherapy and subcutaneous emphysema in a previous punctured keloid scar has not been reported before. She presented sudden swelling of tissue around the treated site and cutaneous crepitus on palpation; those clinical characteristics are the main and almost pathognomonic of air accumulation in skin and subcutaneous tissue [1, 6–8]. This setting was similar to the classical SE clinical evolution secondary to cryotherapy, where clinical manifestations are evident within the first 24 hours [1, 9]. Other signs for this condition are erythema and bubbles mixed with serohematic exudate; those are consequence of the vasodilatation and inflammatory process [6, 7].

The clinical diagnosis in our patient was obvious because of the history of cryotherapy before the onset of symptoms. However, sometimes diagnosis of SE can be challenging because it could have an atypical presentation [4, 6, 7]. X-ray, ultrasonography, computed tomography scan, or magnetic resonance imaging can be used to confirm the diagnosis in patients. In these imaging studies, abnormal gas accumulation in soft tissues is seen [4, 7]. Histopathology of the dermatosis is characterized by the separation of collagen bundles in the dermis without mucin deposits or inflammation in the clear spaces and focal fragmentation of adipocyte cell membranes in the subcutaneous tissue. Nevertheless, skin biopsy is not required for diagnosis [7].

Because of its lethal condition, the most important differential diagnosis is SE caused by gas gangrene [7, 8], which is mainly caused by* Clostridium* species [8, 10]. It can be suspected by history of preceding trauma, extensive destruction of tissue with foul smell, local heat, pain, and systemic signs and symptoms such as fever and malaise [6–8, 10]. This condition shows no spontaneous recovery; thus culture from tissue material and blood must be done to synergize antibiotics and surgical treatment [6, 8, 10]. Other SE causes should also be excluded such as factitious subcutaneous emphysema, dental or endotracheal procedures, respiratory and gastrointestinal tract disease, and loosely sutured wounds [5, 9]. Angioedema and hematoma may look like SE; thus these conditions need to be excluded, too [7].

Our patient recovered spontaneously, so no extra treatment was necessary. This clinical evolution is similar to SE secondary to other dermatologic procedures, where manifestations used to disappear in the next 12 to 96 hours [6, 8, 9]. Sometimes conservative management with rest, support measures, and follow-up visits are the unique necessary treatment [7–9]. However, it has been described that insufflation may be manually forced out from the affected tissue by local and gentle pressure to the edematous area [1, 6]. There is not enough evidence to use low dose steroids and antibiotics [7].

Prognostic is excellent; no further complications of SE secondary to cryotherapy have been reported. It typically has self-limiting evolution, with prompt resolution and without permanent damage or relapses [1, 4, 6]. To prevent SE after cryotherapy we propose to use different cryosurgery techniques when cutaneous barrier is damaged, in atrophic skins and in thin cutaneous areas, especially in case of elderly patients. In those situations cotton swab technique may be preferred instead of spray nozzle.

Through this adverse experience we also confirm the importance of the order of combined therapies for dermatoses such as keloids. In the case of combined use of cryosurgery and other intralesional drugs, cryotherapy must be applied before the preceding interventions; in this way the risk of SE can be avoided.
